# Supplementary figures and images for: Bacterial Communities of Forest Soils along Different Elevations: Diversity, Structure, and Functional Composition with Potential Impacts on CO2 Emission
Source: Microorganisms. 2022 Apr 1;10(4):766. doi: 10.3390/microorganisms10040766 (PMC9032212; doi:10.3390/microorganisms10040766)

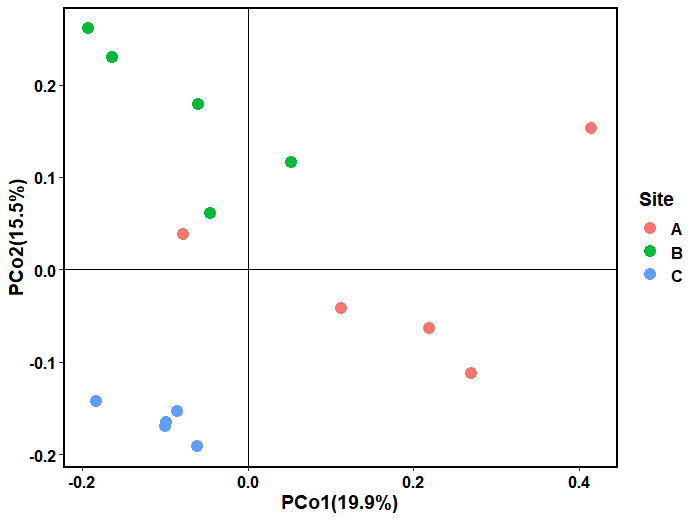

Supplement: Supplementary file 1 [file microorganisms-10-00766-s001.zip › FigureS1 OTU_PCoa.tiff]

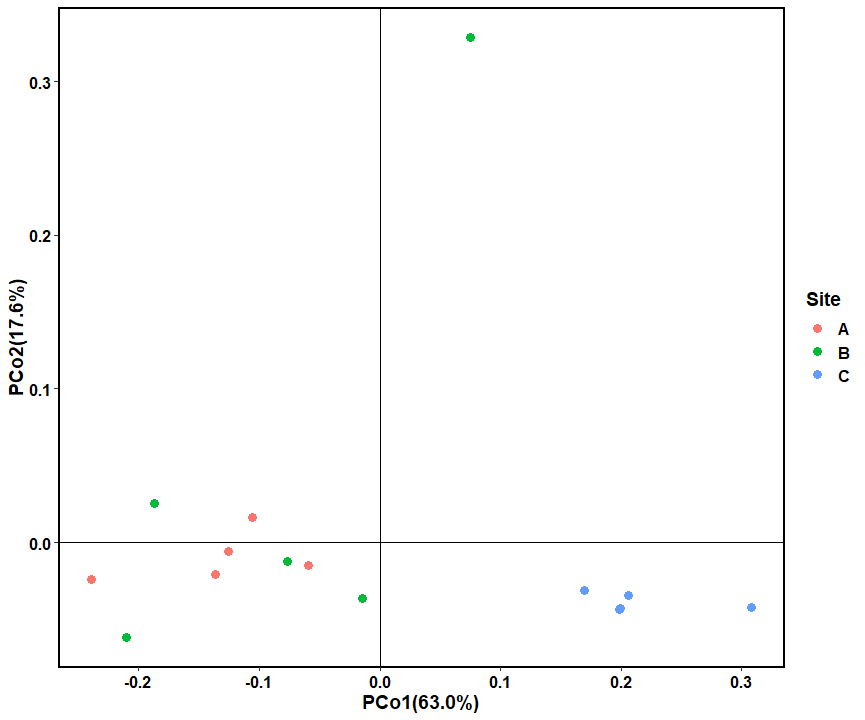

Supplement: Supplementary file 1 [file microorganisms-10-00766-s001.zip › FigureS2 function_PCoa.tiff]

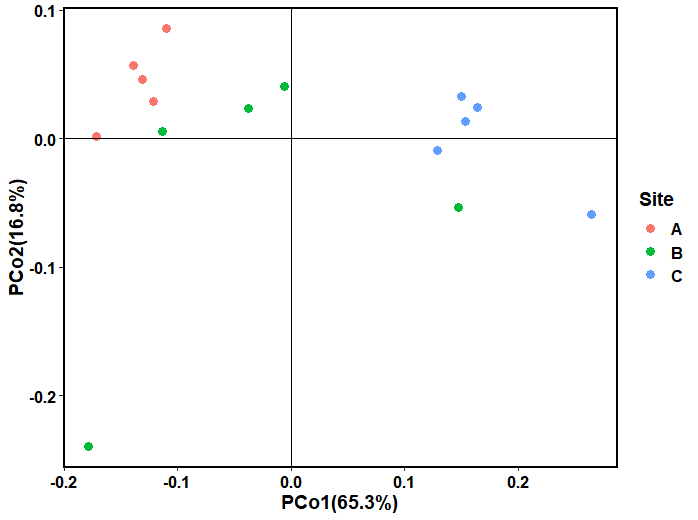

Supplement: Supplementary file 1 [file microorganisms-10-00766-s001.zip › FigureS3 degration_PCoa.tiff]
